# Development of a rapid and visual detection method for *Rickettsia rickettsii* combining recombinase polymerase assay with lateral flow test

**DOI:** 10.1371/journal.pone.0207811

**Published:** 2018-11-26

**Authors:** Yong Qi, Yinxiu Shao, Jixian Rao, Wanpeng Shen, Qiong Yin, Xiaoling Li, Hongxia Chen, Jiameng Li, Wenwen Zeng, Shulong Zheng, Suyun Liu, Yuexi Li

**Affiliations:** 1 Huadong Research Institute for Medicine and Biotechniques, Nanjing, Jiangsu, China; 2 China Pharmaceutical University, Nanjing, Jiangsu, China; Johns Hopkins University, UNITED STATES

## Abstract

**Objectives:**

*Rickettsia rickettsii* is the causative agent of Rocky Mountain spotted fever, which is the most severe spotted fever group (SFG) rickettsiosis. Developing a simple and reliable detection method is required.

**Methods:**

A detection method for *R*. *rickettsii* was established based on a recombinase polymerase amplification (RPA) assay and the lateral flow (LF) test. A specific target sequence was screened, and corresponding primers and probes were designed, synthesized, and screened for establishing an RPA assay with high amplification efficiency. Reagent concentrations, amplification time, and loading volume for strip development were optimized. The detection limit, analytic sensitivity and specificity were evaluated.

**Results:**

A rapid, visual, sensitive and specific method for the detection of *R*. *rickettsii* based on RPA and the LF test was successfully established. The novel method had a limit of detection of 10 to 50 copies/reaction without recognizing other organisms. Analytical sensitivity and specificity were ≥90% and 100%, respectively, as evaluated by animal and simulative human samples.

**Conclusions:**

Using the established method, detection could be completed in 30 min with visually detectable results by the naked eye, without requirement of any instrument except a constant temperature equipment. The technique shows superior detection performance and is promising for wide use in the field as well as resource-limited areas for *R*. *rickettsii* detection.

## Introduction

*Rickettsia rickettsii* is the causative agent of Rocky Mountain spotted fever (RMSF), which is the most severe spotted fever group (SFG) rickettsiosis [1,2]. From its first appearance in the Bitter Root Valley in Montana in 1873 to the year 1903, probably 200 cases of the severe type have occurred, with 70% to 80% being fatal [3]. Although doxycycline is available for the treatment of RMSF, fatal cases are still reported yearly, and case fatality rates approach 7% in some regions to which the disease is highly endemic in the United States [4–6]. In Mexico, case fatality rates are as high as >30%, as estimated from recent outbreaks [7–9]; in Brazil, with a general case-fatality rate of approximately 20 to 40% [10,11], this rate may reach 80% in a few areas [12]. Delayed diagnosis and/or treatment have been highlighted as one of the factors that increase the risk of dying from RMSF [5,12].

Currently, the diagnosis of RMSF relies on serological and molecular tests, with immunofluorescence assay (IFA) being the gold standard. However, serologic assays are frequently negative during the acute phase of illness [6], due to antibodies being formed only at 1–2 weeks post infection. Molecular tests include conventional polymerase chain reaction (PCR), nested PCR, and real-time quantitative PCR (RT-qPCR). PCR and nested PCR are not reliable for use in acute-phase blood samples, which may have very few organisms [6]. In addition, they may need another step of sequencing to confirm the species, which is time consuming. RT-qPCR has been proved to be more sensitive and useful [1,6]. However, it requires a fluorescence quantitation instrument, which is expensive and not suitable for areas with limited instruments and infrastructure.

Recently, a new isothermal amplification method termed recombinase polymerase amplification (RPA) assay was developed [13]. This method, relying on three proteins, i.e. a recombinase, a polymerase, and a single strand binding protein, can achieve amplification in 5–20 min at a constant temperature of around 37°C, with a limit of detection lower than 10 copies/reaction [13]. Moreover, when a biotin-labeled reverse primer, a carboxyfluorescein (FAM)-labeled probe, and the nfo (an endonuclease) enzyme are introduced in the RPA assay, the amplified product is labeled by both biotin and FAM, and can be detected visually using the lateral flow (LF) test. Combination of RPA with LF (RPA-LF) is suitable for developing a simple, rapid, sensitive and specific method for the detection of *R*. *rickettsii*, and would be useful in resource-limited areas. In the present study, such a detection method for *R*. *rickettsii* was established and evaluated.

## Methods

### Ethics statement and DNA preparation

Genomic DNA samples of *R*. *rickettsii* (strain Sheila Smith), *Rickettsia heilongjiangensis* (strain 054), *Rickettsia sibirica* (strain 246), and *Coxiella burnetii* (strain Xinqiao) were kindly provided by Professor Bohai Wen at Beijing Institute of Microbiology and Epidemiology. Genomic DNA from *Orientia tsutsugamushi* (strain Sanjie), *Staphylococcus aureus* and *Streptococcus suis* (type II) was extracted with a QIAamp Blood and Tissue Mini DNA kit (Qiagen, CA, USA). For quality control, genomic DNA solutions of *R*. *rickettsii*, *C*. *burnetii*, *R*. *heilongjiangensis*, *R*. *sibirica*, *O*. *tsutsugamushi*, *S*. *aureus*, and *S*. *suis* were quantitated by RT-qPCR as described previously [14–20] to be 10^5^ to 10^8^ copies/μL.

DNA from *R*. *rickettsii*-infected mouse spleens was also provided by Professor Bohai Wen. Briefly, 4 to 6-week-old female C3H/HeN mice were infected with a sub-lethal dose of purified *R*. *rickettsii*. On day 5 post infection, mice were sacrificed, and spleens were separated. DNA in 10 mg of spleen tissue was purified with a QIAamp Blood and Tissue Mini DNA kit (Qiagen), and the concentrations (copies/μL) of *R*. *rickettsii* DNA were determined by RT-qPCR as described previously [14].

*R*. *rickettsii* DNA-spiked human samples were prepared to simulate samples from RMSF patients for lack of actual patient specimens. Briefly, blood samples were collected into K2E (EDTA) Blood Collection Tubes (BD Vacutainer, Franklin Lakes, NJ, USA) from the cubital vein of healthy volunteers and centrifuged at 2000×g for 10 min to prepare plasma. Various concentrations of genomic DNA of *R*. *rickettsii* were mixed with 200 μL of plasma to obtain DNA-spiked samples. DNA from the spiked samples or normal plasma were extracted with a QIAamp Blood and Tissue Mini DNA kit (Qiagen). Concentrations of *R*. *rickettsii* DNA in DNA-spiked samples were quantified by RT-qPCR as described previously [14].

All animal experiments and the use of human blood samples were approved by the Ethics Committee of Huadong Research Institute for Medicine and Biotechniques. Mice were well cared for during housing in the facility, and all efforts were made to minimize suffering. The experiments were carried out in accordance with the approved guidelines. Consent forms were signed by the patients.

### Specific sequence screening and positive plasmid construction

Genomic DNA sequences of *R*. *rickettsii* (strain Sheila Smith, accession no.: CP000848.1) were used for alignment with those of several species of spotted fever group Rickettsia, including *Rickettsia conorii* (strain Malish 7, accession no.: NC_003103), *R*. *sibirica* (strain 246, accession no.: NZ_AABW01000001), and *Rickettsia akari* (strain Hartford, accession no.: NC_009881), using the MAUVE 2.3.1 software to screen a sequence specific to *R*. *rickettsii*. The screened sequence was then analyzed using Nucleotide BLAST online (https://blast.ncbi.nlm.nih.gov/Blast.cgi) to evaluate its specificity.

Positive recombinant plasmid containing the target sequence was constructed by conventional molecular biology methods as described elsewhere [21]. Briefly, the target sequence was amplified by polymerase chain reaction (PCR) with primers listed in Table 1 and the genomic DNA of *R*. *rickettsii* as a template, using a Premix Taq Version 2.0 kit (Takara, Dalian, China). The primers were designed to contain *Eco*R I and *Bam*H I restriction sites, respectively. The amplification product was analyzed by agarose gel electrophoresis followed by purification with a QIAquick Gel Extraction kit (Qiagen). The pUC19 plasmid was extracted from *Escherichia coli* carrier cells. Both the purified target gene and plasmid were digested by *Eco*R I and *Bam*H I, analyzed by agarose gel electrophoresis, purified with QIAquick Gel Extraction kit (Qiagen), and ligated with a DNA ligation kit (Takara). The recombinant plasmid was transformed into competent *E*. *coli* cells and spread on solid Luria-Bertani (LB) medium containing ampicillin. The recombinant plasmid in positive bacterial colonies was purified, digested by both *Bam*H I and *Eco*R I, and analyzed by agarose gel electrophoresis. The recombinant plasmid was also sequenced (GenScript, Nanjing, China). The concentration of the purified positive plasmid was measured by Nanodrop one (Thermo Fisher Scientific, Shanghai, China). The copy number of the purified plasmid was determined as follows: concentration (copies/μL) = 6.02×10^23^×plasmid concentration (ng/μL)×10^−9^/(DNA length×660).

**Table 1:** Primer and probe sequences for PCR and RPA assay.

Primers or Probe		Nucleotide sequence (5’-3’)
PCR primers	FP	AAGGATCCAAACAGGAAATTAATTCT
	RP	GCGAATTCTAGTTTATCGTATTCTTGA
RPA forward primers	Rrf53	CTAGCAATAATCTGTGTTATTTGATAAAAT
	Rrf84	AAATCTAAAAGAAAGGGAAAGTGTATGAGT
	Rrf120	AACGCTAAGTTTTACATACCATTGGTGTCA
RPA reverse primers	Rrr220	Biotin-AACACAAATACTAAAGAACACTGAGATATT
	Rrr252	Biotin-GCTCAGATATAATATTGGTAGTTATAGCTA
	Rrr277	Biotin-ATCAAAGCTCTCTTTCTACCATATAGCTCA
	Rrr307	Biotin-AAACTTACTATTATACATAACGCCACAGCA
	Rrr379	Biotin-ACAAAAGATGCAAAAACAACTCCTTT
RPA probe	Rrprobe	FAM-TACATACCATTGGTGTCACTACTTGGTGTA[THF]TCATTTATTTACTAA-PO4

### Primer and probe design, and screening

Primers and the probe, including 3 forward primers, 5 reverse primers, and 1 probe, were designed manually (Table 1) according to design principles provided by TwistDX Limited (manufacturer of RPA related kit, Cambridge, UK). The probabilities of formation of hairpin, self-dimer, false priming, and cross dimer of primers and the probe were evaluated with the Primer premier 5.0 software to ensure their availability. The reverse primers were labeled with biotin at the 5’ end. The probe was labeled with carboxyfluorescein (FAM) at the 5’ end, with a block group of phosphate at the 3’ end, and a base analog tetrahydrofuran (THF) inserted between the 30^th^ and 31^st^ bases. All primers and the probe were synthesized by GenScript (Nanjing, China).

Forward and reverse primers were combined to generate various combinations, and the best group was screened using a preliminary RPA reaction system recommended in the commercial TwistAmp RPA nfo kit (TwistDx Limited). Briefly, 2.1 μL of forward primer (10 μM), 2.1 μL of reverse primer (10 μM), 0.6 μL of probe (10 μM), 1 μL of template (positive plasmid or control pUC19 plasmid, at a concentration of 1×10^4^ copies/μL), 12.2 μL of DNase- and RNase-free water, and 29.5 μL of rehydration buffer (provided in the kit) were mixed to rehydrate the enzyme pellet of lyophilized recombinase, polymerase, and single-strand binding protein (provided in the kit). Then, 2.5 μL of MgAc (280 mM) was added to initiate the reaction followed by incubation at 37°C for 20 min. After the reaction, 5 μL of the amplified product, which had both FAM and biotin labeling at the ends, was diluted in 95 μL of Tris-buffered saline, and a Milenia Genline Hybridetect-1 (MGH) strip (Milenia Biotec GmbH, Gieben, Germany) was developed by immersing the sample pad of the strip in the dilution for 3 to 5 min. Results were judged visually by the naked eye. A positive result was determined when both test (T) and control (C) lines developed, indicating the existence of sequences labeled with both FAM and biotin in the product. Only C line occurrence indicated a negative result; if the C line does not develop, the strip should be replaced. All the reactions were performed in duplicate.

Sequence conservation between the best forward and reverse primers was analyzed by aligning with the corresponding sequences of all 11 strains of *R*. *rickettsii* with the DNAman software (version 5.2.2). The 11 included strains were: Iowa isolate Small Clone (accession no.: CP018914.1), Iowa isolate Large Clone (accession no.: CP018913.1), Morgan (accession no.: CP006010.1), Hauke (accession no.: CP003318.1), Hino (accession no.: CP003309.1), Arizona (accession no.: CP003307.1), Colombia (accession no.: CP003306.1), Brazil (accession no.: CP003305.1), Sheila Smith (accession no.: CP000848.1), R (accession no.: CP006009), and Hlp#2 (accession no.: CP003311.1).

### Optimization of reaction conditions

Various concentrations of the reverse primer and probe (Table 2) were used in the RPA-LF method as mentioned above, and the best concentration group was determined via the developing strips. In addition, various amplification times (10 min, 15 min, and 20 min) for RPA reaction and loading volumes (1 μL, 2 μL, or 5 μL) of the amplified product for strip development were evaluated in the RPA-LF method to obtain an optimized detection system. The positive plasmid or control pUC19 plasmid at a concentration of 1×10^4^ copies/μL was used as a template in the reaction.

**Table 2:** Optimization of concentrations of the reverse primer Rrr379 and the probe Rrprobe in the RPA-LF reaction system.

Group No.	Concentrations (Rrr379 & Rrprobe)	Results
1	10 μM & 10 μM	-
2	10 μM & 5 μM	++
3	10 μM & 2.5 μM	++
4	5 μM & 10 μM	-
5	5 μM & 5 μM	++
6	5 μM & 2.5 μM	-
7	2.5 μM & 10 μM	-
8	2.5 μM & 5 μM	-
9	2.5 μM & 2.5 μM	+

Groups with false positive or false negative results were classified as negative (-); the remaining groups with darkest T line in the experimental strip were classified as excellent (++), and those with modest dark T line in the experimental strip were considered to be good (+).

### Evaluation of the limits of detection

Serial dilutions of positive plasmids were performed to concentrations of 1×10^4^, 1×10^3^, 1×10^2^, 1×10^1^, and 1×10^0^ copies/μL, respectively, and used as templates in the optimized RPA-LF method to evaluate the limit of detection of positive plasmids.

Serial dilutions of the genomic DNA of *R*. *rickettsii* from around 10^4^ to 10^0^ copies/μL were used as templates in the optimized RPA-LF method to evaluate the limit of detection of the genomic DNA. These dilutions were assessed by RT-qPCR to evaluate the accuracy of concentrations.

The limit of detection was determined as the concentration of the highest dilution yielding a positive detection result. Each positive plasmid or genomic DNA dilution was evaluated in duplicate.

### Specificity and sensitivity evaluation

To evaluate the specificity and discrimination ability from phylogenetically related bacteria or unrelated bacteria of the optimized RPA-LF method, genomic DNA samples from various pathogens, including *R*. *rickettsii*, *C*. *burnetii*, *O*. *tsutsugamushi*, *R*. *heilongjiangensis*, *R*. *sibirica*, *S*. *aureus*, and *S*. *suis*, were mixed with equal volumes of DNA from human plasma, and used as templates in the optimized RPA-LF method. DNA from human plasma only was used as a control template. All reactions were performed in duplicate.

To evaluate the sensitivity and specificity of the method, DNA from *R*. *rickettsii*-infected or uninfected mouse spleens, and from *R*. *rickettsii* DNA-spiked human plasma or human plasma only, respectively, were used as templates in the optimized RPA-LF method. The DNA concentrations of animal samples were around 10^3^ to 10^4^ copies/μL as determined by RT-qPCR; those of simulative human samples were approximately 50 to 500 copies/μL. The analytic sensitivity and specificity in the detection of infected mouse samples or simulative human samples were determined. All reactions were carried out in duplicate.

## Results

### Screening of the specific target sequence

A target sequence of *R*. *rickettsii* (strain Sheila Smith, accession no. CP000848.1) from around 724500 to 727000 was screened by genome alignment with the MAUVE software (S1 Fig). Considering the RPA method performs better with short amplification products of around 200 bp, we selected a sequence of 688 bp from base 725922 to 726609 as the final target sequence to perform the RPA assay. The 688 bp sequence was then analyzed with Nucleotide BLAST online (https://blast.ncbi.nlm.nih.gov/Blast.cgi) to evaluate its specificity, and no significantly similar sequence was found in species other than *R*. *rickettsii* in GenBank, indicating its high specificity (S2 Fig). A gene encoding the hypothetical protein A1G_04230 (from base 724653 to 724874), which was previously screened as a target for RT-qPCR detection of *R*. *rickettsii*, was also contained in the sequence from base 724500 to 727000 [1,6]. The 688 bp target sequence in the present study just followed the 3’ end of the A1G_04230 gene, and contained a gene encoding the hypothetical protein A1G_04240.

### Positive plasmid construction

The target sequence was amplified by PCR and recombined with the pUC19 plasmid to generate the recombinant plasmid pUC19-688. The constructed plasmid was verified by double enzyme digestion. As shown in S3 Fig, two fragments of about 2700 bp and 700 bp were obtained, respectively, in agreement with the sizes of pUC19 and the target sequence, respectively. The recombinant plasmid was sequenced to be exactly the same as the corresponding target sequence, indicating the successful establishment of the positive plasmid.

### Designing and screening of primers and the probe for RPA

**Fig 1:**
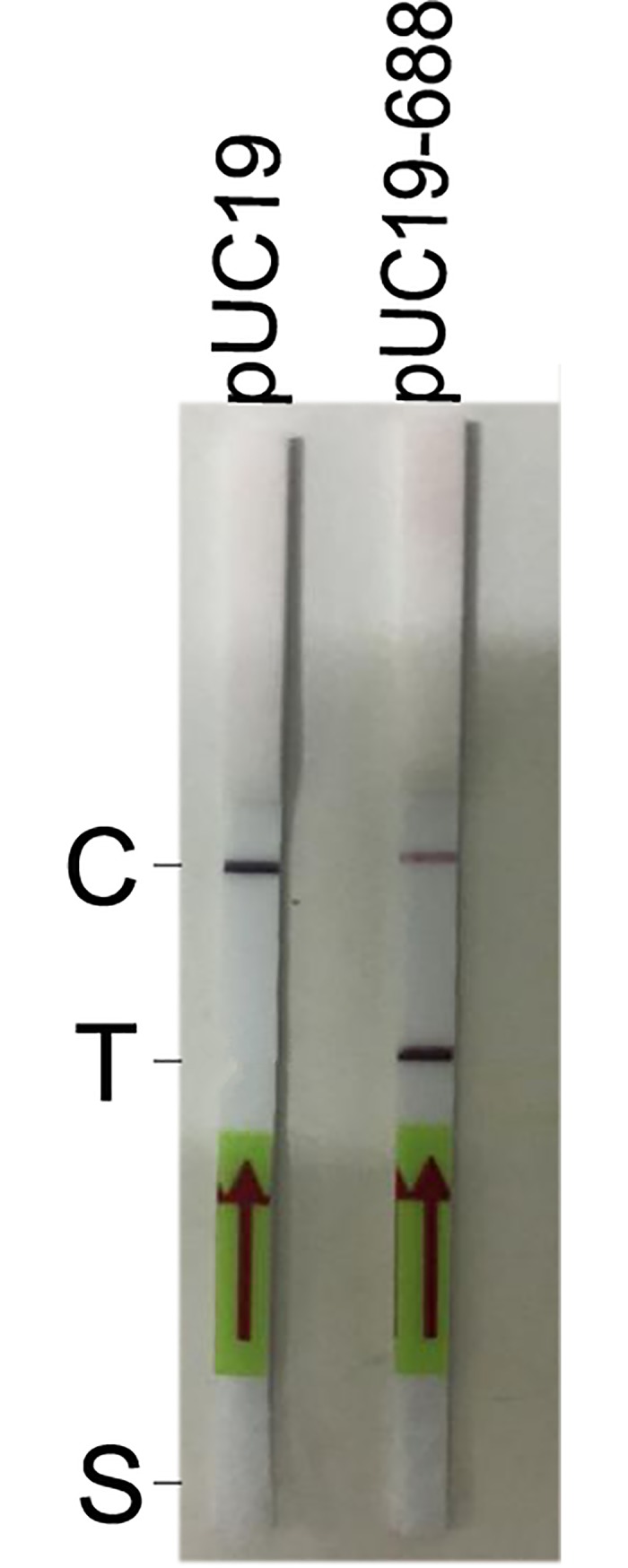
Results of RPA-LF with the Rrf53 and Rrr379 primer combination. The pUC19 (1×10^4^ copies/reaction) or pUC19-688 (1×10^4^ copies/reaction) plasmid was used as a template. C, C line; T, T line; S, sample pad.

Three forward primers, five reverse primers, and one probe were designed and synthesized for screening. Actually, in the preliminary study, only four reverse primers were designed, and the combination of three forward and four reverse primers did not yield good results. Another reverse primer (Rrr379) was designed and combined with each forward primer. As shown in Fig 1, the Rrf53 and Rrr379 primer combination, with an intensive dark band on the T line of the experimental strip (with pUC19-688 as template) and no band on the T line of the control strip (with pUC19 as template), met the standard of good result and was identified as the best combination of primers.

### Conservation analysis of the target sequence between best primers

**Fig 2:**
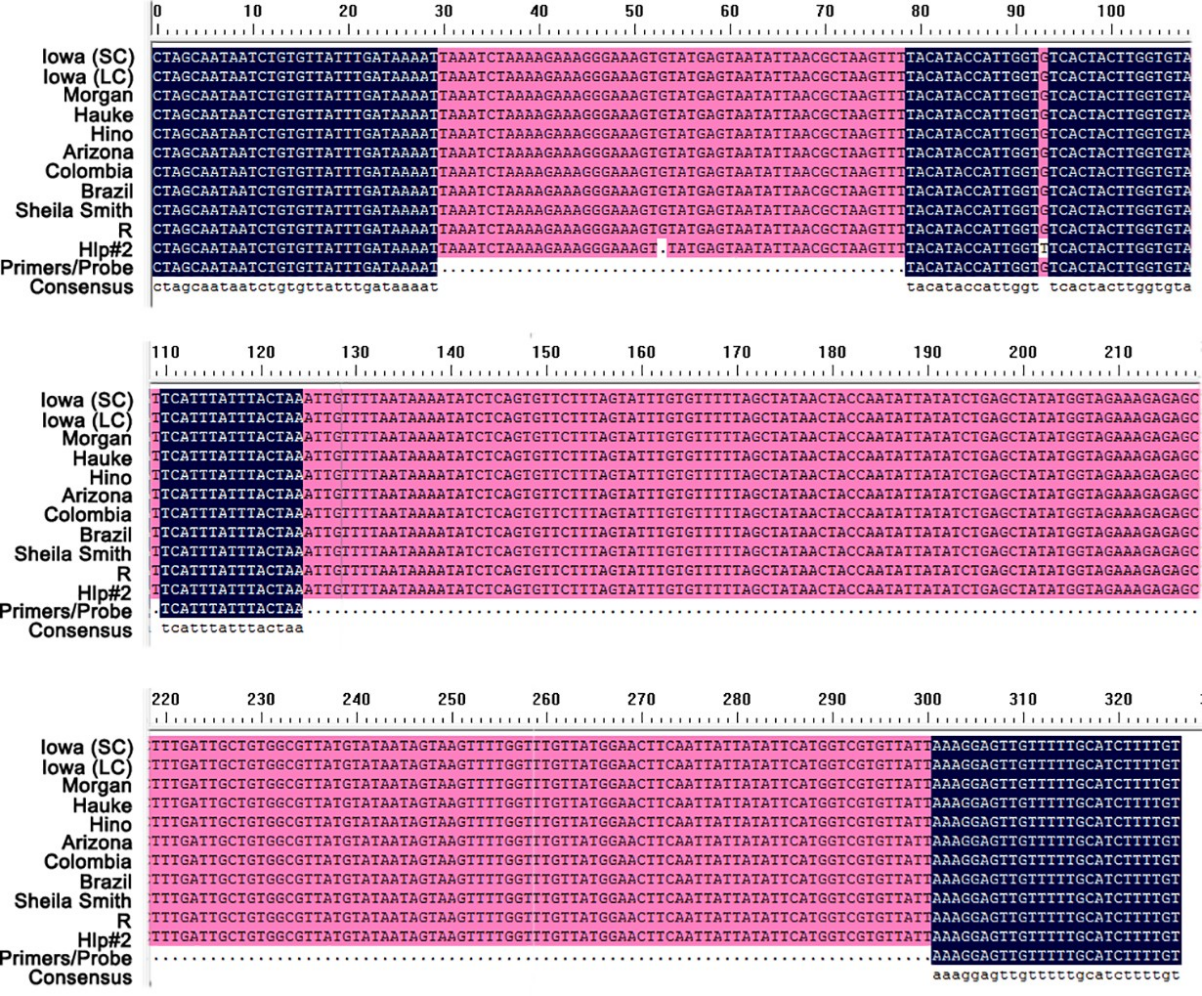
Alignment of the target sequence between the forward Rrf53 and reverse Rrr379 primers among various *Rickettsia rickettsii* strains. Strains used were: Iowa isolate Small Clone (accession no.: CP018914.1), Iowa isolate Large Clone (accession no.: CP018913.1), Morgan (accession no.: CP006010.1), Hauke (accession no.: CP003318.1), Hino (accession no.: CP003309.1), Arizona (accession no.: CP003307.1), Colombia (accession no.: CP003306.1), Brazil (accession no.: CP003305.1), Sheila Smith (accession no.: CP000848.1), R (accession no.: CP006009), and Hlp#2 (accession no.: CP003311.1).

As a target sequence, conservation is of the same importance as specificity. Therefore, the target sequence between primers Rrf53 and Rrr379 was aligned with the corresponding sequence in all 11 strains of *R*. *rickettsii* with the DNAman software. As shown in Fig 2, sequences from all strains except strain Hlp#2 were identical, and only one base in the probe mismatched with that of strain Hlp#2, indicating the target sequence was conserved, and the established method was suitable for the detection of almost all strains of *R*. *rickettsii*.

### Optimization of the RPA-LF detection method

**Fig 3:**
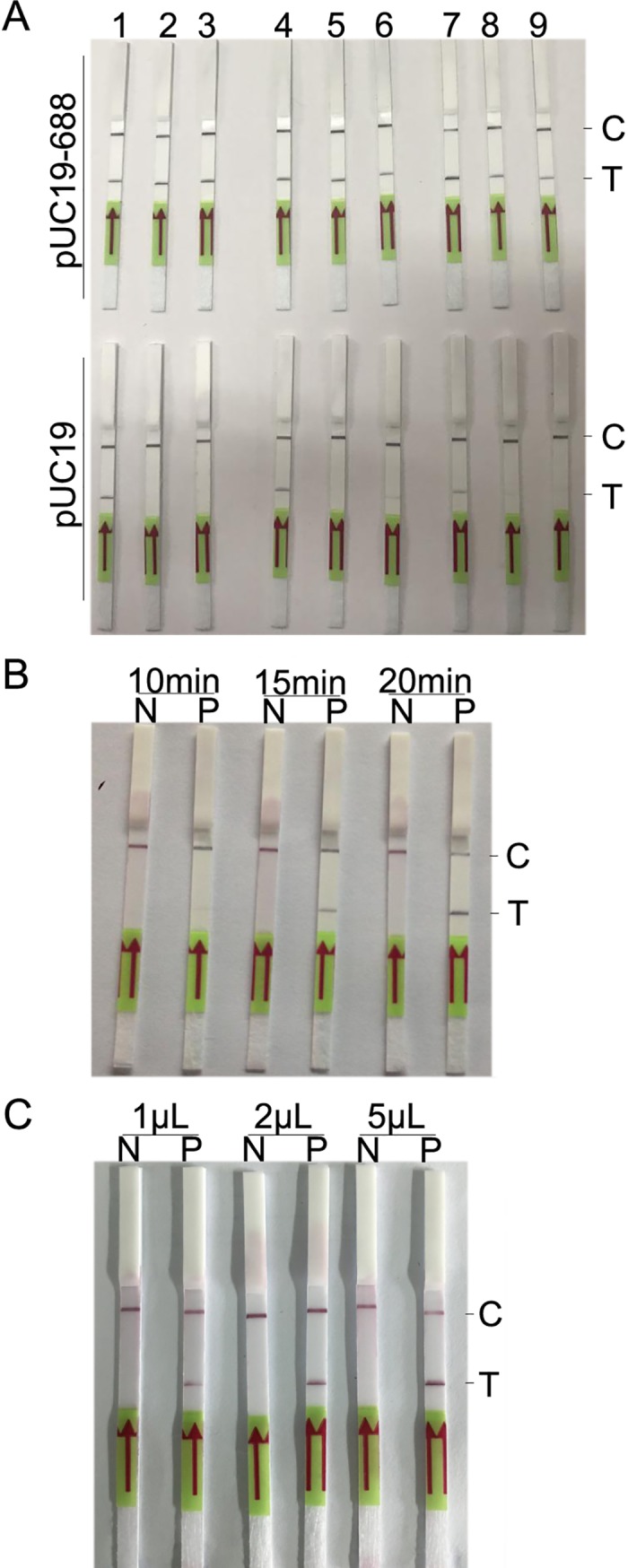
**Optimization of reverse primer and probe (A) concentrations, reaction time of the RPA assay (B), and loading volume of the amplified product on strips (C).** Groups 1 to 9 in (A) are detailed in Table 2. N, pUC19 (1×10^4^ copies/reaction) as a template; P, pUC19-688 (1×10^4^ copies/reaction) as a template; C, C line; T, T line.

Concentrations of reverse primer Rrr379 and the probe (Rrprobe) in the RPA assay were optimized. Based on experimental (pUC19-688 as template) and control (pUC19 as template) strips, groups 2 (10 μM of Rrr379 and 5 μM of Rrprobe), 3 (5 μM of Rrr379 and 5 μM of Rrprobe), and 5 (10 μM of Rrr379 and 2.5 μM of Rrprobe) performed well (Fig 3A and Table 2). Group 3 with 5 μM of Rrr379 and 5 μM of Rrprobe was selected for subsequent experiments on account of economic cost.

The reaction time of the RPA assay and the loading volume of amplified products on strips were optimized. Only a faint band developed on the T line after 10 min of amplification, while the color became darker with increasing amplification time (Fig 3B). The loading volume showed similar results, with the color becoming darker with increasing loading volume (Fig 3C). To achieve a high analytical sensitivity, an amplification time of 20 min and a loading volume of 5 μL were selected in the optimized RPA-LF detection method.

### Evaluation of the limit of detection of the RPA-LF method

The limit of detection of the RPA-LF method was evaluated using both the positive plasmid pUC19-688 and the genomic DNA of *R*. *rickettsii*.

**Fig 4:**
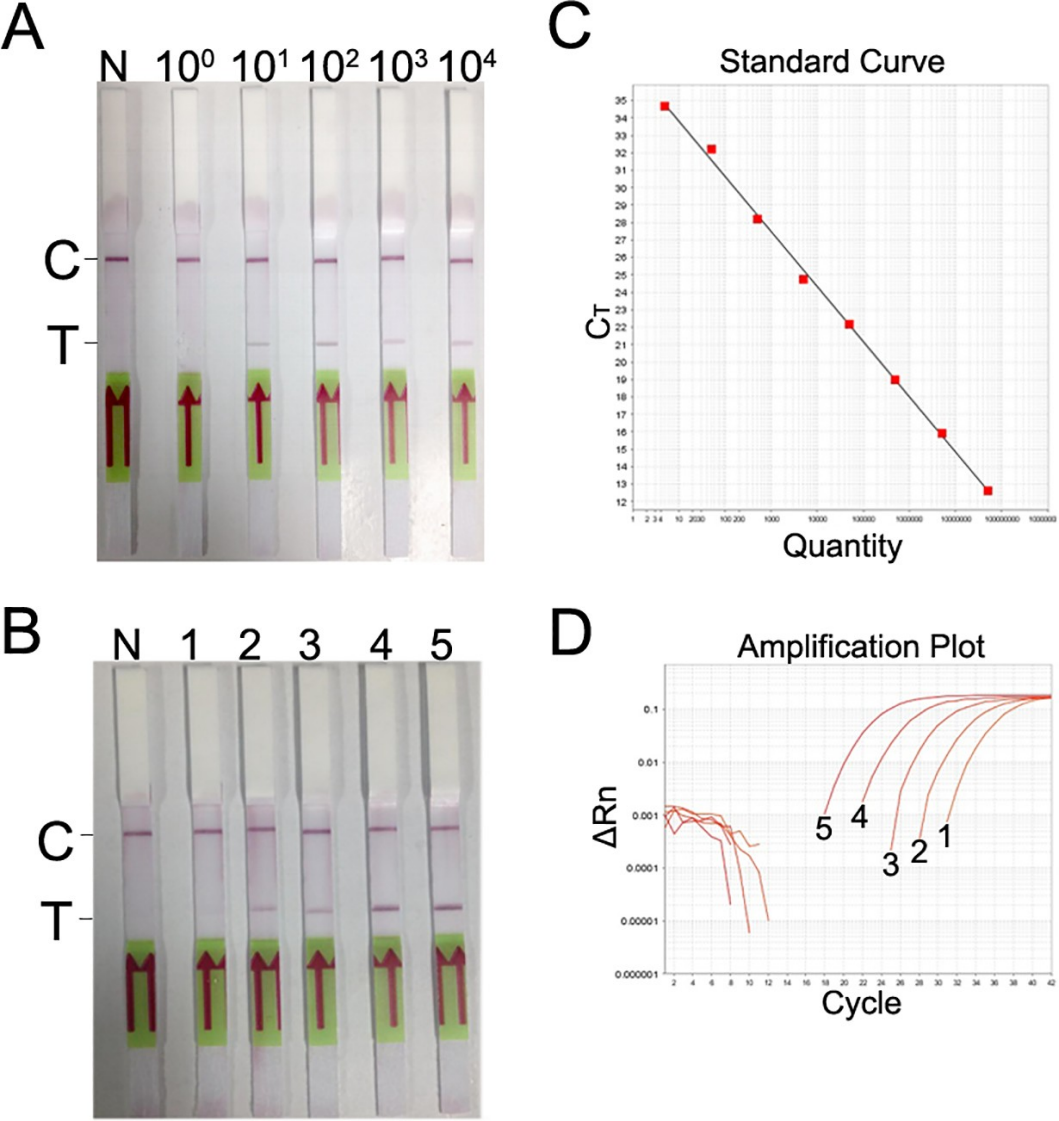
**Evaluation of the limit of detection of the RPA-LF method for the positive plasmid pUC19-688 (A) or genomic DNA of *R*. *rickettsii* (B).** The genomic DNA concentrations of dilutions 1 to 5 in (B) were determined by RT-qPCR (C, D) to be 6.0×10^0^, 5.0×10^1^, 5.5×10^2^, 7.3×10^3^, and 8.7×10^4^ copies/μL, respectively.

For the positive plasmid pUC19-688, a concentration of as low as 10 copies/μL was detectable (Fig 4A), indicating a limit of detection of the RPA-LF for the plasmid of 10 copies/reaction.

A series of dilutions of the genomic DNA of *R*. *rickettsii* were assessed, and dilutions 2 to 5, with concentrations of 5×10^1^, 5.5×10^2^, 7.3×10^3^, and 8.7×10^4^ copies/μL, respectively, as determined by RT-qPCR, could be visually detected on strips (Fig 4B, 4C and 4D), while dilution no. 1 (6 copies/μL determined by RT-qPCR) was not detectable, indicating the established RPA-LF method could detect as low as 50 copies/reaction or lower (concentrations between 6 to 50 copies/reaction were not accurately determined in this study).

### Specificity and sensitivity of the RPA-LF method

**Fig 5:**
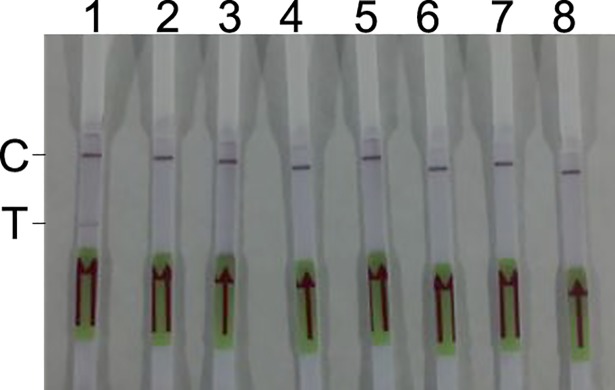
Specificity of the RPA-LF method. Strips 1 to 8 used genomic DNA samples from *R*. *rickettsii* (7×10^3^ copies/reaction), *C*. *burnetii* (3×10^5^ copies/reaction), *O*. *tsutsugamushi* (1×10^6^ copies/reaction), *R*. *heilongjiangensis* (1×10^6^ copies/reaction), *R*. *sibirica* (7×10^6^ copies/reaction), *S*. *aureus* (8×10^7^ copies/reaction), and *S*. *suis* (6×10^7^ copies/reaction), and human plasma DNA, respectively, as templates to evaluate the RPA-LF method.

To evaluate whether the established method could recognize other bacteria, genomic DNA samples from several bacteria were assessed. As shown in Fig 5, T lines did not develop when the RPA-LF method was used for DNA from *R*. *heilongjiangensis*, *R*. *sibirica*, *O*. *tsutsugamushi*, *C*. *burnetii*, *S*. *aureus*, *S*. *suis*, and human plasma; meanwhile, the T line developed for *R*. *rickettsii* DNA.

**Fig 6:**
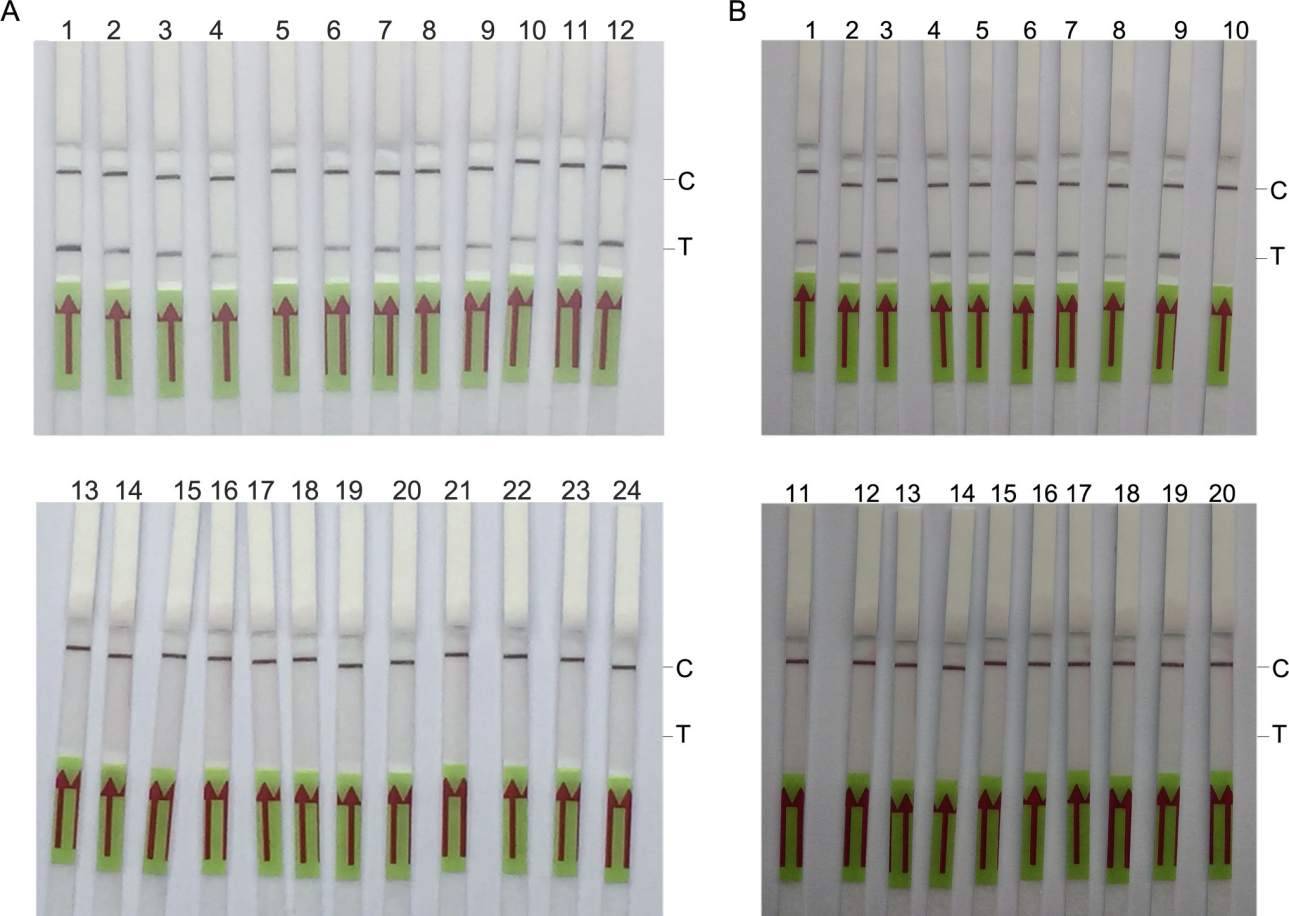
Analytical sensitivity and specificity of the established method with DNA from *R*. *rickettsii*-infected (strips 1 to 12 in A) or uninfected mouse samples (strips 13 to 24 in A), and DNA-spiked human plasma (strips 1 to 10 in B) or human plasma only (strips 11 to 20 in B).

The analytical sensitivity and specificity of the established method were evaluated with *R*. *rickettsii*-infected mouse samples or DNA-spiked human specimens. As shown in Fig 6A, all *R*. *rickettsii*-infected mouse samples, which contained around 10^3^ to 10^4^ copies/μL of genomic DNA as determined by RT-qPCR, were detected by the RPA-LF method, while none of the uninfected mouse samples was recognized, indicating that both sensitivity and specificity were 100% in mouse samples. Meanwhile, the RPA-LF method could detect 9 out of 10 DNA-spiked human samples (around 50 to 500 copies/μL of genomic DNA) and none of the 10 normal human specimens, indicating an analytical sensitivity of 90% and a specificity of 100% in simulative human samples.

## Discussion

In the first round of primer screening, no primer groups worked well, with either T lines developing in both experimental and control groups (false positive) or no development (false negative). Actually, based on the development mechanism of the T line on the strip, any molecule labeled with both biotin and FAM could develop the T line. Therefore, if the reverse primer (with biotin labeling) and the probe (with FAM labeling) form a dimer, a false positive result would occur. Therefore, we screened the best primer combination and optimized concentrations of the reverse primer and the probe in this study. Another reverse primer Rrr379 was designed to prevent any dimers to be formed with the probe, and worked well with the forward primer Rrf53. In the concentration optimization procedure, false positive results were also observed at a high concentration of the probe (10 mM; lanes 1, 4, and 7 in Fig 3A). It is possible that a high concentration of the probe made it easier to form a dimer with the reverse primer.

In the RPA-nfo assay, two rounds of amplification existed for one reaction. The first round used the forward and biotin-labeled reverse primers to amplify the existing target sequence; the amplified products were used as templates for the second round. The FAM-labeled probe annealed with the amplified product lost its 3’ block by nfo enzyme cutting in the THF site, and would function with biotin-labeled reverse primer as a primer pair in the subsequent amplification. A FAM- and biotin-labeled amplified product would develop the T line on the MGH strip. Considering the reverse primer and the probe directly influenced the development of the MGH strip, reverse primer and probe concentrations were optimized but not that of the forward primer.

The optimized RPA-LF method could detect 50 copies/reaction of genomic DNA, making it useful for assessing patient samples in the acute phase. The limit of detection could be lower than 50 copies/reaction of genomic DNA, considering that 10 copies of plasmid/reaction were detected. However, the specific value between 6 to 50 copies/reaction was not accurately determined for the genomic DNA.

The specificity of the method was evaluated using genomic DNA samples from various bacteria. *R*. *heilongjiangensis* and *R*. *sibirica* belong to SFG Rickettsia; *O*. *tsutsugamushi* belongs to Rickettsieae; *C*. *burnetii* is traditionally classified to Rickettsiales because of similar biological characteristics. The above bacteria are phylogenetically related to *R*. *rickettsii*, unlike *S*. *aureus* and *S*. *suis*. Bacterial genomic DNA samples from all the above bacteria were not detected by the RPA-LF method. In addition, specificity was 100% as evaluated with limited animal samples and simulative human specimens. Analytical sensitivity was found to be 100% and 90% for animal and simulative human samples, respectively. Considering the DNA copy numbers in simulative human samples were around or just a little higher than the limit of detection, it is not unexpected that one of these samples was not detected by the RPA-LF method. Actually, no patient samples were available in our laboratory, and only simulative human samples of DNA-spiked plasma were used here. As previously described, detection of rickettsial DNA using patient blood/serum specimens in the acute phase does not result in clinical sensitivity as good as that obtained with skin biopsy or eschar samples because of the transient character of bacteremia [22–24]. Therefore, clinical sensitivity should be tested using various clinical specimens in the future although analytical sensitivity was good in this study.

The RPA-LF method was less sensitive in the present study than RT-qPCR, which could detect as low as 5 copies/reaction of the positive plasmid or genomic DNA. However, the RPA-LF method could be completed in less than 30 min, with the RPA assay consuming 20 min and the LF test taking less 5 min. In addition, the method does not require any expensive instrument, and can even be implemented with human body temperature without instrument [25]. Compared with RT-qPCR, the established RPA-LF method is more suitable for field studies and basic medical units with limited resources.

A limitation of the established RPA-LF method may be that an internal control was not used to eliminate potential inhibitors in samples. This may limit the actual use of this novel technique. Introducing a control sample to be tested together with the positive plasmid as a parallel specimen may solve the above limitation and improve clinical performance. Moreover, developing a universal detection method for all SFG Rickettsia pathogens to cooperate with the established RPA-LF method for *R*. *rickettsii* detection may be more useful in clinical diagnosis.

In conclusion, we successfully established a simple, sensitive and specific method for the detection of *R*. *rickettsii* based on a RPA assay and the LF test. The novel method could detect all *R*. *rickettsii* strains. It had a detection limit of 10 to 50 copies/reaction without recognizing other organisms like *R*. *heilongjiangensis*, *R*. *sibirica*, *O*. *tsutsugamushi*, *C*. *burnetii*, *S*. *aureus*, and *S*. *suis*, and was suitable for mouse or human samples. The analytical sensitivity and specificity of the method were ≥90% and 100%, respectively, for animal and simulative human samples. The RPA-LF method can be completed in 30 min without requiring any experiment instrument except a constant temperature equipment, with visually detectable results by the naked eye. It is therefore promising for wide use in the field and resource-limited areas, although actual clinical samples are needed to further evaluate this technique.

## Supporting information

S1 Fig Alignment of genomic DNAs of four pathogens of spotted fever group Rickettsia using the MAUVE 2.3.1 software. Genomes of *R*. *rickettsii* (strain Sheila Smith, accession no.: CP000848.1), *R*. *conorii* (strain Malish 7, accession no.: NC_003103), *R*. *sibirica* (strain 246, accession no.: NZ_AABW01000001), and *R*. *akari* (strain Hartford, accession no.: NC_009881) were assessed.
(TIF)

S2 Fig Alignment of the 688-bp target sequence in the GenBank database using the Nucleotide BLAST software online. (TIF)

S3 Fig Agarose gel electrophoresis analysis of recombinant pUC19-688 digested by *Bam*H I and *Eco*R I. M, DNA marker; P, digested pUC19-688. Size is indicated on the left.
(TIF)
