# Supplementary figures and images for: Development of a rapid and visual detection method for Rickettsia rickettsii combining recombinase polymerase assay with lateral flow test
Source: PLoS One. 2018 Nov 26;13(11):e0207811. doi: 10.1371/journal.pone.0207811 (PMC6257923; doi:10.1371/journal.pone.0207811)

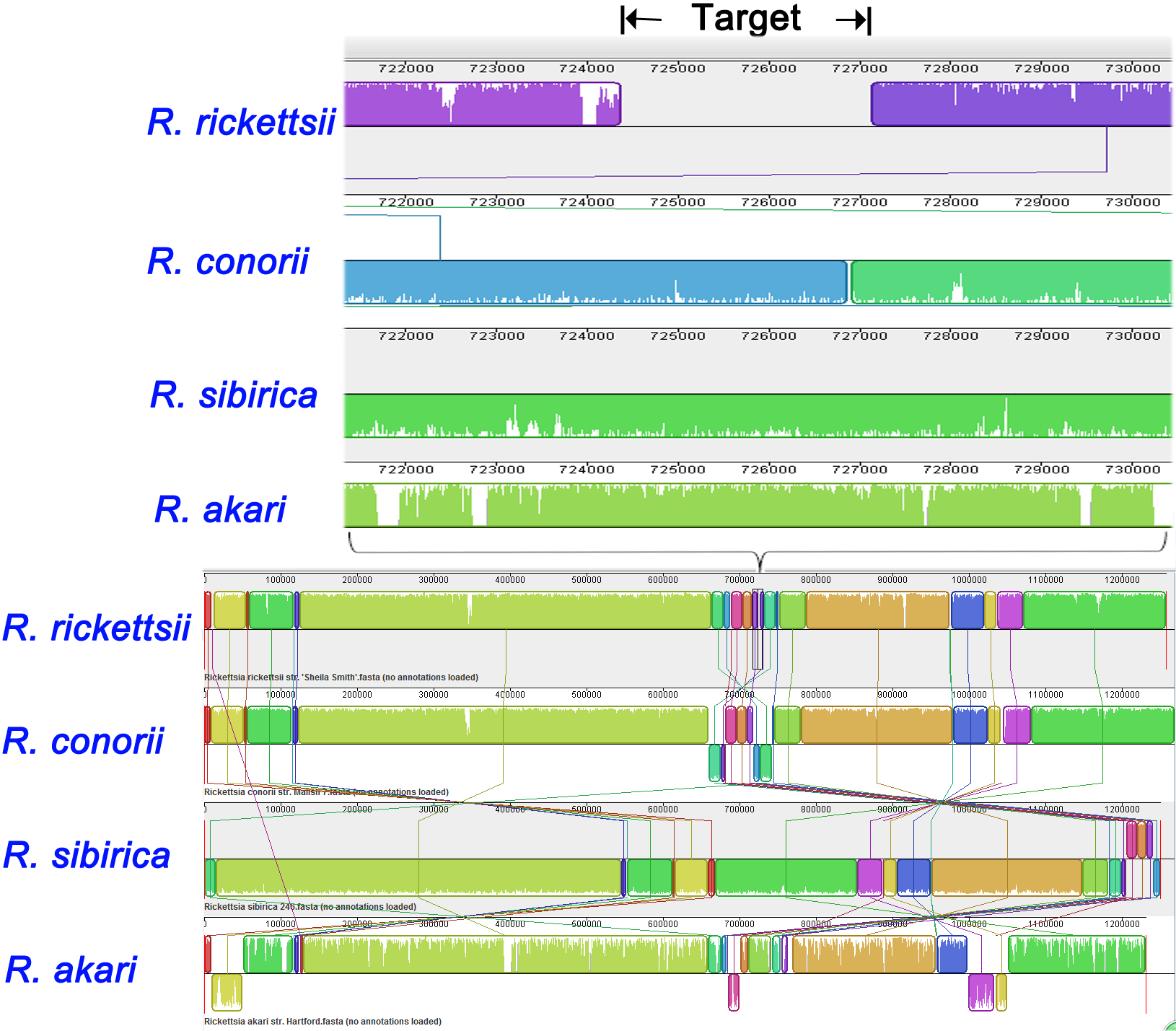

Supplement: S1 Fig — Genomes of R. rickettsii (strain Sheila Smith, accession no.: CP000848.1), R. conorii (strain Malish 7, accession no.: NC_003103), R. sibirica (strain 246, accession no.: NZ_AABW01000001), and R. akari (strain Hartford, accession no.: NC_009881) were assessed. (TIF) [file pone.0207811.s001.tif]

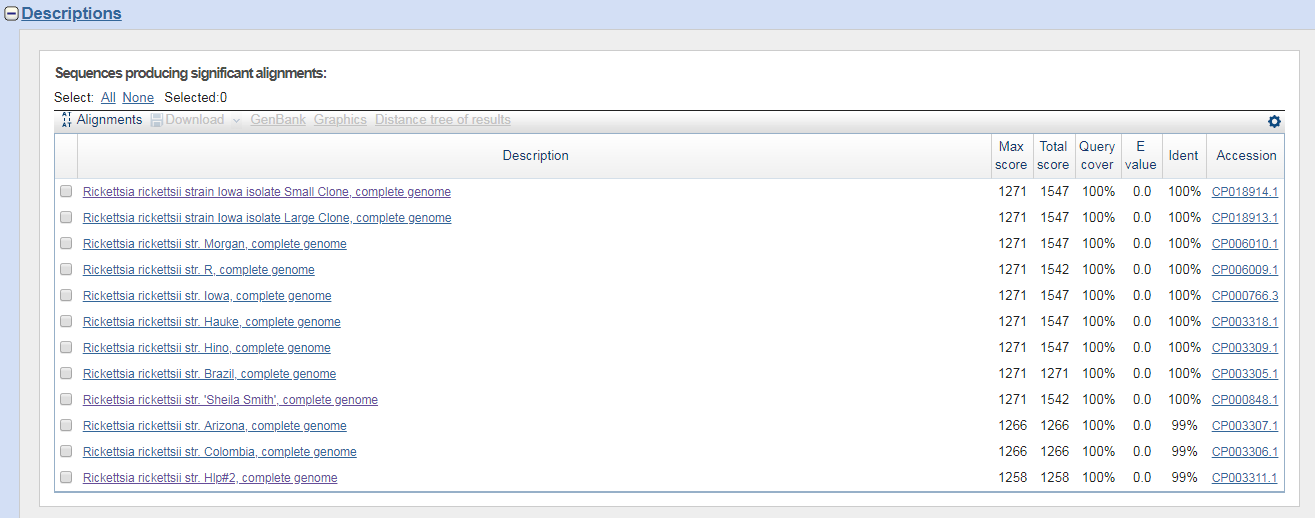

Supplement: S2 Fig — (TIF) [file pone.0207811.s002.tif]

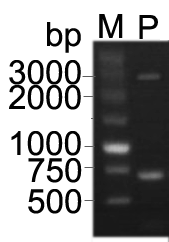

Supplement: S3 Fig — M, DNA marker; P, digested pUC19-688. Size is indicated on the left. (TIF) [file pone.0207811.s003.tif]
